# A Cautionary Tale: A Case Report Describing a Benign Parotid Oncocytoma Diagnosed as Metastatic Squamous Cell Carcinoma on Fine Needle Aspirate

**DOI:** 10.7759/cureus.50853

**Published:** 2023-12-20

**Authors:** Danjel Miladinovic, Jodie Trautman, Takako Yabe

**Affiliations:** 1 General Surgery, Prince of Wales Hospital, Sydney, AUS; 2 General Surgery, Wollongong Hospital, Wollongong, AUS

**Keywords:** fine needle aspirate, metastatic squamous cell carcinoma, case report, diagnostic challenge, salivary gland, parotid oncocytoma

## Abstract

Parotid oncocytoma is a rare salivary gland tumour. Management does not require surgical intervention if diagnosed on a preoperative biopsy.

A 64-year-old man presented with a parotid mass diagnosed as mcSCC on fine-needle aspiration cytology (FNAC). Surgical histopathology following parotidectomy demonstrated an oncocytoma of the parotid gland.

Parotid oncocytomas are rare; therefore, diagnosis can be difficult. An MRI, CT, and US-guided biopsy are required for diagnosis. Ultrasound-guided core biopsy (UGSB) is more sensitive and specific when compared to FNAC for diagnosing malignant tumours. Diagnoses of benign salivary gland tumours on biopsy remain challenging.

The aim of this article is to highlight the difficulty of diagnosing salivary gland tumours. We further aim to outline the contributing features that lead to this misdiagnosis and suggest steps to circumvent it in the future.

This report describes the challenges in diagnosing salivary gland tumours and outlines the contributing features of this misdiagnosis. We add to the literature an additional case of a parotid oncocytoma.

## Introduction

Salivary gland tumours are uncommon, representing only 2% of all human tumours; rarer still are parotid oncocytomas, with their incidence representing 1% of salivary gland tumours [1]. Oncocytomas are more prevalent in the sixth and seventh decades of life and have a higher incidence in women [2]. Unlike some other salivary gland tumours, oncocytomas do not typically require surgical intervention.

In Australia, the most common malignant tumour of the parotid gland is metastatic cutaneous squamous cell carcinoma (mcSCC), which often requires aggressive multimodal treatment [3]. The diagnosis can be challenging due to the morphological similarities shared between benign and malignant tumours [4]. Fine-needle aspiration cytology (FNAC) is a valuable diagnostic tool, with a reported accuracy rate ranging from 85-96%, a sensitivity of 75-100%, and a specificity of 81-100% [5]. The literature describes few cases of parotid oncocytoma and no cases of oncocytoma have been reported following a FNAC diagnosis of mcSCC.

The significance of an accurate diagnosis is imperative to obviate surgical intervention and its associated risks. The aim of this article is to highlight the difficulty of diagnosing salivary gland tumours and further suggest steps to avoid future misdiagnosis.

We describe the case of a 64-year-old man who presented with a parotid mass. This case report presents a unique scenario in which a patient’s FNAC suggested mcSCC but subsequent surgical histopathology revealed an oncocytoma of the parotid gland.

## Case presentation

A 64-year-old man presented with a two-month history of a painless mass in his right parotid gland. He had no associated local compressive, invasive, or constitutional symptoms. His past medical history included no previous skin cancer, high alcohol consumption, and being a non-smoker.

**Figure 1:**
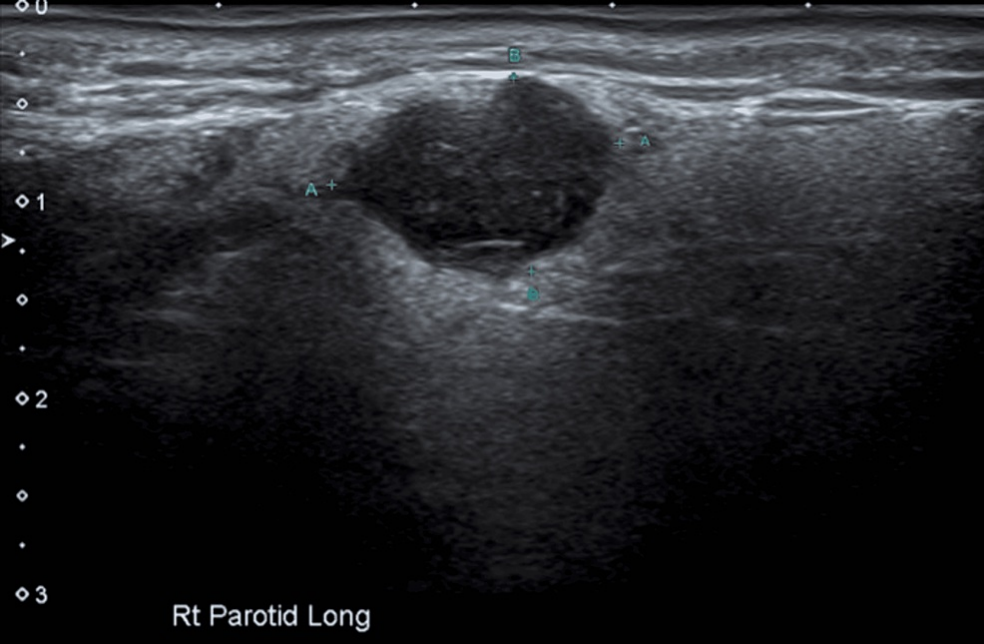
Ultrasound of right parotid gland showing a 14 mm × 11 mm × 12 mm cystic lesion

Neck ultrasound (US), magnetic resonance imaging (MRI), and positron emission tomography (PET) were completed in the workup. The US showed a cystic lesion within the parotid gland, measuring 14 mm × 11 mm × 12 mm, reported to be in keeping with a salivary tumour or abnormal lymph node (Figure 1).

**Figure 2:**
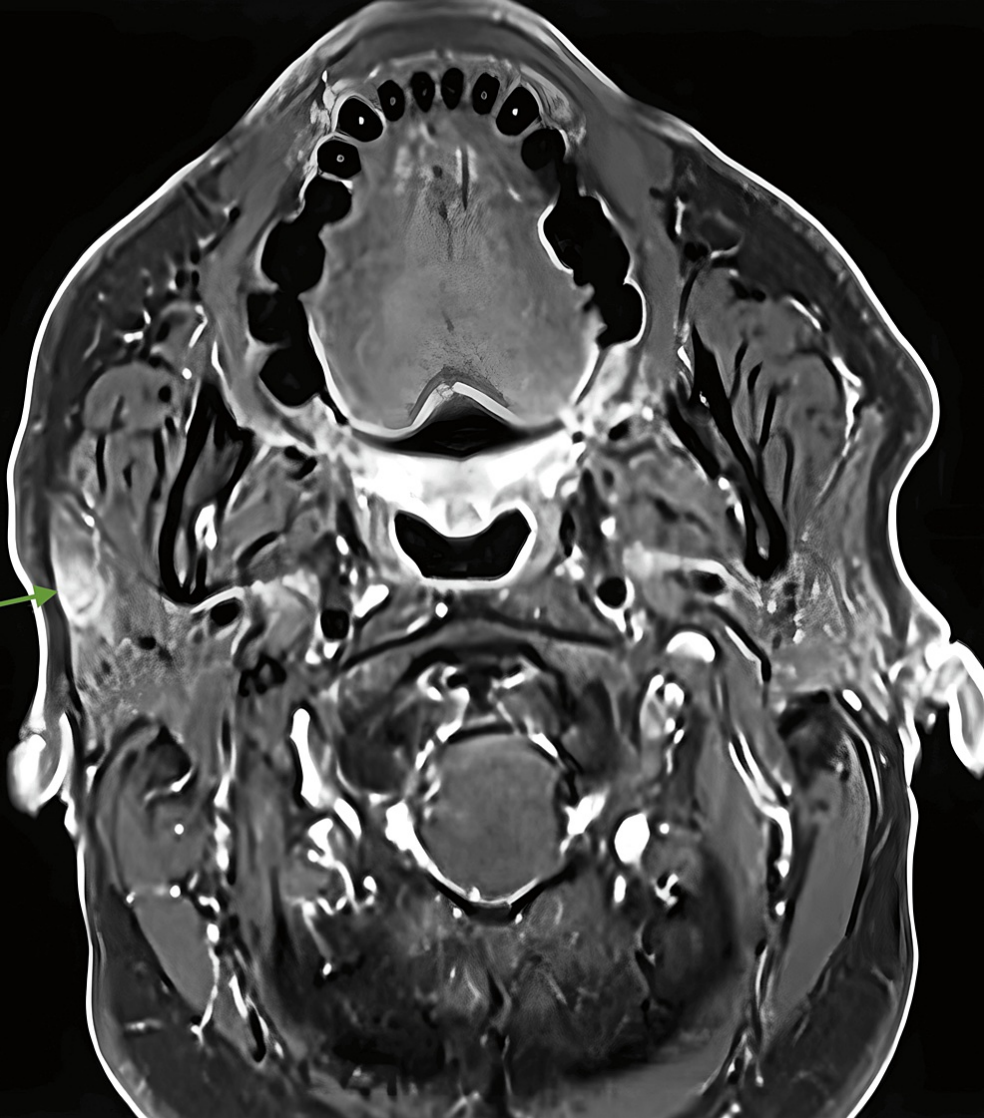
A MRI of the right parotid gland showing 10 mm × 5 mm indeterminant lentiform-shaped lesion

MRI showed a 10 mm × 5 mm indeterminant lentiform-shaped lesion in the right parotid, possibly compatible with SCC. There was no definitive evidence of pathological lymphadenopathy in the neck (Figure 2).

**Figure 3:**
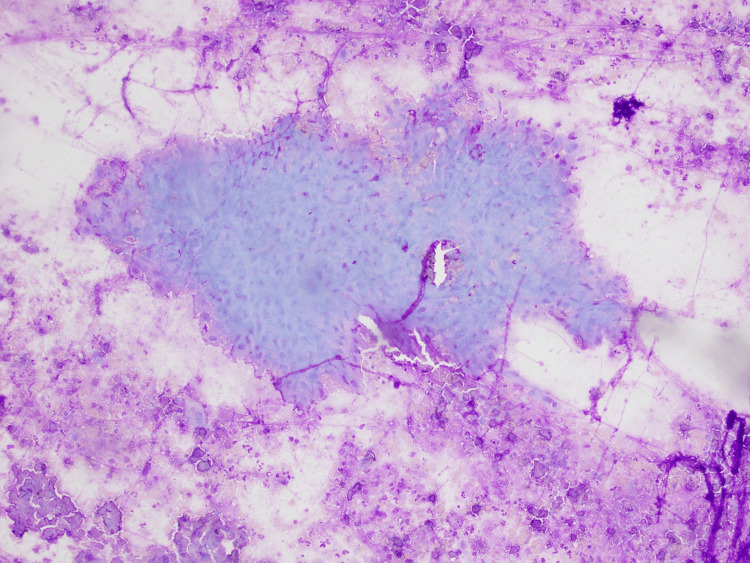
Microscopy from FNAC showing oncocytic cells with evidence of necrosis

PET indicated mild hypermetabolic uptake in the right inferior parotid (SUV max 2.8) with no evidence of distant metastases. Microscopy from FNAC reported oncocytic cells with evidence of necrosis (Figure 3).

**Figure 4:**
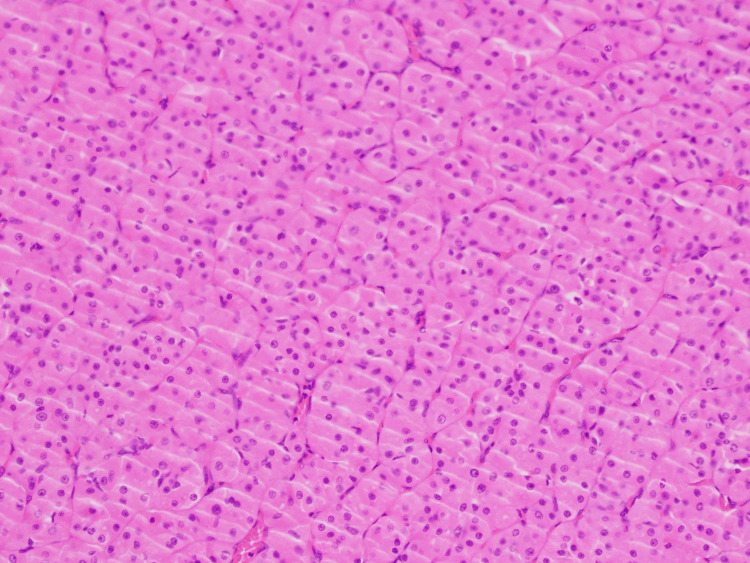
Final surgical histopathology showing oncocytoma/oxyphilic adenoma of the parotid gland

Immunohistochemistry was challenging to interpret due to these degenerate changes. P40 and CR5/6 were reported as focally positive, suggesting squamous cell morphology. The patient was discussed at a Head and Neck Oncology Multidisciplinary Team (MDT) meeting. The decision was made to proceed with a right-sided superficial parotidectomy with selective neck dissection. His post-operative course was uneventful. The final histopathological analysis of the surgically resected specimen confirmed the presence of an oncocytoma/oxyphilic adenoma of the parotid (Figure 4), in contrast to the initial FNAC diagnosis of mcSCC.

## Discussion

Parotid oncocytomas pose a diagnostic challenge due to their rarity, and it is crucial to accurately diagnose benign tumours to avoid unnecessary surgical interventions and associated risks. Various imaging modalities play a role in the diagnosis of salivary gland tumours.

Oncocytomas are reported as homogeneously hypoechoic ovoid masses, typically well-defined, lobulated, and highly vascular on US [2,6]. MRI oncocytoma is hypodense on T1 and isointense on T2 [7]. MRI of mcSCC in a large case series commonly showed large lobulated tumours with ill-defined margins, heterogenous enhancement, and necrosis [8]. While a helpful adjunct in the workup, PET can be hypermetabolic in both benign and malignant diseases of the parotid [9].

Ultrasound-guided core biopsy (UGCB) is a valuable tool for diagnosing salivary gland lesions, with a reported sensitivity of 83-100% and a specificity of 100% compared to surgical histopathology [10,11]. In comparison to this, FNAC has a reported sensitivity of 82%, a specificity of 95%, and a 70% diagnostic concordance compared to surgical histopathology for malignant lesions [12]. Notwithstanding, its sensitivity for the diagnosis of oncocytoma is as low as 29% [13]. Oncocytoma usually demonstrates a large cytoplasm-to-nucleus ratio filled with mitochondria (Figure 3) [14]. The discordance with our case was the necrosis combined with degenerate cells (Figure 4) and the immunohistochemistry suggestive of malignancy likely from squamous cell origin.

A MDT meeting allows the collaboration of surgeons, pathologists, oncologists, and allied health members to discuss appropriate patient management. This collaboration remains a crucial tool in the workup and diagnosis of head and neck tumours, with particular emphasis on pathologists and their role in cytopathological interpretation. Diagnosing salivary gland tumours is challenging because of the variety of histological patterns made by matrix production of myoepithelial cells, the similar morphology between tumours and the heterogeneity of cellular differentiation [15]. Many salivary gland neoplasms (benign and malignant) present as oncocytic lesions on cytopathology, including Warthin tumours, pleomorphic adenoma, oncocytoma oncocytic carcinoma, acinic cell carcinoma, mucoepidermoid carcinoma, cystedemoma, cystedenocarcinoma, and metastases [16,17]. In this case, the degenerate oncocytes demonstrated evidence of nuclear atypia. Thus, the samples were reported as showing evidence of metaplasia and malignancy [1].

In this case, phenotyping is helpful as it adds further information to the indeterminant findings on cytology. Malignant oncocytic salivary gland tumours have varying immunohistochemical markers depending on the tumour type. In this case, the specific immunohistochemical markers p40 and CK5/6 were positive, suggesting squamous cell differentiation, leading to the conclusion that the salivary tumour was likely SCC [15,18].

In retrospect, when the features are challenging to interpret, it may be advisable to consider further investigation with a core biopsy. A core biopsy is more sensitive and specific in diagnosing lesions of the parotid, and it may have obviated the need for an invasive surgical procedure in this case.

## Conclusions

This case report highlights the challenges associated with diagnosing parotid oncocytomas, a rare entity in the medical literature. It emphasises the importance of an accurate diagnosis to avoid unnecessary surgical interventions and their associated risks. The initial misdiagnosis of metastatic cutaneous squamous cell carcinoma based on FNAC underscores the limitations of this diagnostic tool. UGCB is suggested as an alternate diagnostic tool to prevent surgical intervention and improve diagnostic approaches, particularly in cases of uncertain FNAC results. By sharing this case, we contribute to the existing body of knowledge on the diagnostic complexities of salivary gland tumours.
